# Novel molecular evidence of population structure in *Anopheles* (*Kerteszia*) *bellator* from Brazilian Atlantic Forest

**DOI:** 10.1590/0074-02760180598

**Published:** 2019-05-13

**Authors:** Kamila Voges, Marcela Possato Correa da Rosa, Betina Westphal-Ferreira, Mario Antonio Navarro-Silva, Carime Lessa Mansur Pontes, André Nóbrega Pitaluga, Carlos José de Carvalho-Pinto, Luísa DP Rona

**Affiliations:** 1 Universidade Federal de Santa Catarina, Centro de Ciências Biológicas, Departamento de Biologia Celular, Embriologia e Genética, Florianópolis, SC, Brasil; 2 Universidade Federal do Paraná, Setor de Ciências Biológicas, Departamento de Zoologia, Curitiba, PR, Brasil; 3 Universidade Federal de Santa Catarina, Centro de Ciências Biológicas, Departamento de Microbiologia, Imunologia e Parasitologia, Florianópolis, SC, Brasil; 4 Fundação Oswaldo Cruz-Fiocruz, Instituto Oswaldo Cruz, Laboratório de Biologia Molecular de Parasitas e Vetores, Rio de Janeiro, RJ, Brasil; 5 Imperial College London, Department of Life Sciences, London, United Kingdom; 6 Instituto Nacional de Ciência e Tecnologia em Entomologia Molecular, Rio de Janeiro, RJ, Brasil

**Keywords:** Anopheles (Kerteszia) Bellator, bromeliad-malaria, Brazilian Atlantic Forest

## Abstract

*Anopheles bellator* is a primary malaria vector in the Atlantic Forest. Partial sequences of *timeless* and *Clock* genes were used to assess the genetic differentiation of five Brazilian populations, which showed strong population structure (e.g. high *F*_*ST*_ values and fixed differences) in all pairwise comparisons between Bahia sample and the others from Paraná, São Paulo and Rio de Janeiro states. Also, the resulting phylogenetic trees clearly grouped the sequences from Bahia in a different cluster with high bootstrap values. Among southern and southeastern populations low levels of genetic differentiation were found suggesting a general stability of the genetic structure.

*Anopheles* (*Kerteszia*) *bellator*, a mosquito species whose immature stages develop in water accumulated in bromeliads, occurs in Trinidad and Tobago and coastal areas spanning eastern Venezuela to southern Brazil.^1^ ^,^ ^2^ Interestingly, it is only considered a main human malaria vector in the two extremes of its distribution area,^3^ ^,^ ^4^ and despite its epidemiological importance, there are few molecular studies concerning this species. Carvalho-Pinto and Lourenço-de-Oliveira^5^ evaluated *An. bellator* isoenzymes in populations from three locations in Brazil [Santa Catarina (SC), São Paulo (SP) and Bahia (BA)], and one from Trinidad Island (Trinidad and Tobago). They concluded that the Trinidad Island population was genetically distant from the Brazilian populations, mostly from BA. Also, regarding the three Brazilian samples, SC and SP were genetically close to each other, and quite distant from BA. Recently, a handful of studies have tried to understand the evolutionary relationship among different species from *Kerteszia* subgenus (including *An. bellator*, *Anopheles cruzii* and *Anopheles homunculus*),^6^ ^,^ ^7^ rather than investigating the genetic diversity between *An. bellator* populations. Therefore, to date, there have been no further investigations regarding the relationship between *An. bellator* populations from Brazil.

From the south of Brazil to the north-east, *An. bellator*, *An. homunculus* and *An. cruzii* are sympatric in the Atlantic Forest.^1^ ^,^ ^8^ However, while *An. homunculus* populations from these regions showed no clear genetic separation ― they were in fact considered a single species ― *An. cruzii* was shown to be a complex of at least two siblings: one major group with broad range dispersion from south and south-east Brazil and a distinct sibling species in BA.^8^ ^,^ ^9^ ^,^ ^10^ ^,^ ^11^ This knowledge raises an interesting question: are *An. bellator* populations highly structured like those from *An. cruzii*; or, like *An homunculus*, is it a species without significant genetic differentiation in its wide range distribution? To answer this question and to better understand the genetic variability and divergence among *An. bellator* populations, partial fragments of *timeless* and *Clock* genes were evaluated in five Brazilian locations: Ilha do Mel (PR), Cananéia (SP), Abraão and Sítio Forte (RJ) and Camacan (BA). *Clock* and *timeless* loci are involved in the circadian rhythms of insects,^12^ and have previously been shown to be good molecular markers to define different species in the *An. cruzii* complex.^10^ ^,^ ^11^ ^,^ ^13^ ^,^ ^14^

## MATERIALS AND METHODS

**Fig. 1:**
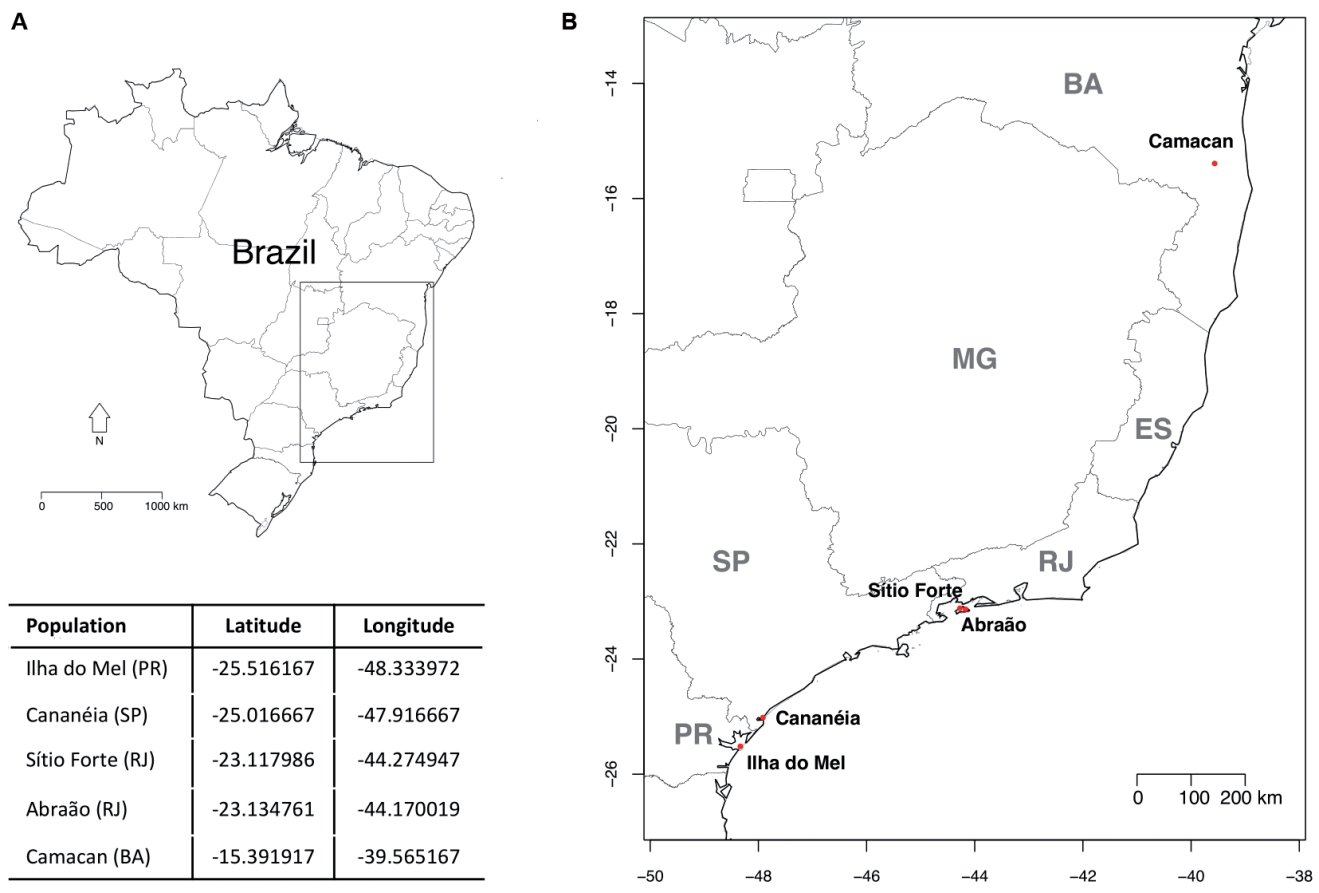
*Anopheles bellator* collection sites in the Brazilian Atlantic Rainforest. (A): map of Brazil. (B): magnification of the black box from (A) displaying the sites where *An. bellator* samples were collected. The x and y axes drawn in the map (B) display the longitude and latitude, respectively.

*Sampling sites* - The mosquitoes used in this study were captured along the Brazilian Atlantic Forest at the following localities: Ilha do Mel (PR), Cananéia (SP), Abraão and Sítio Forte (RJ), and Camacan (BA) (Fig. 1). The maps found in Fig. 1 were made using the packages *maptools*, *maps* and *GISTools* ^15^ ^,^ ^16^ ^,^ ^17^ from R Software version 3.3.2.^18^

*An. bellator* adults and immatures were obtained, treated and preserved as described in de Rezende Dias et al.^14^ The immatures were collected in bromeliads located on rocks, in Restinga areas. Species identification was carried out according to Consoli and Lourenço-de-Oliveira.^2^ The detailed field collection information, such as sex and the life stage in which the samples were collected (adult or larvae), of each specimen is shown in Supplementary data (Table I).

**Fig. 2:**
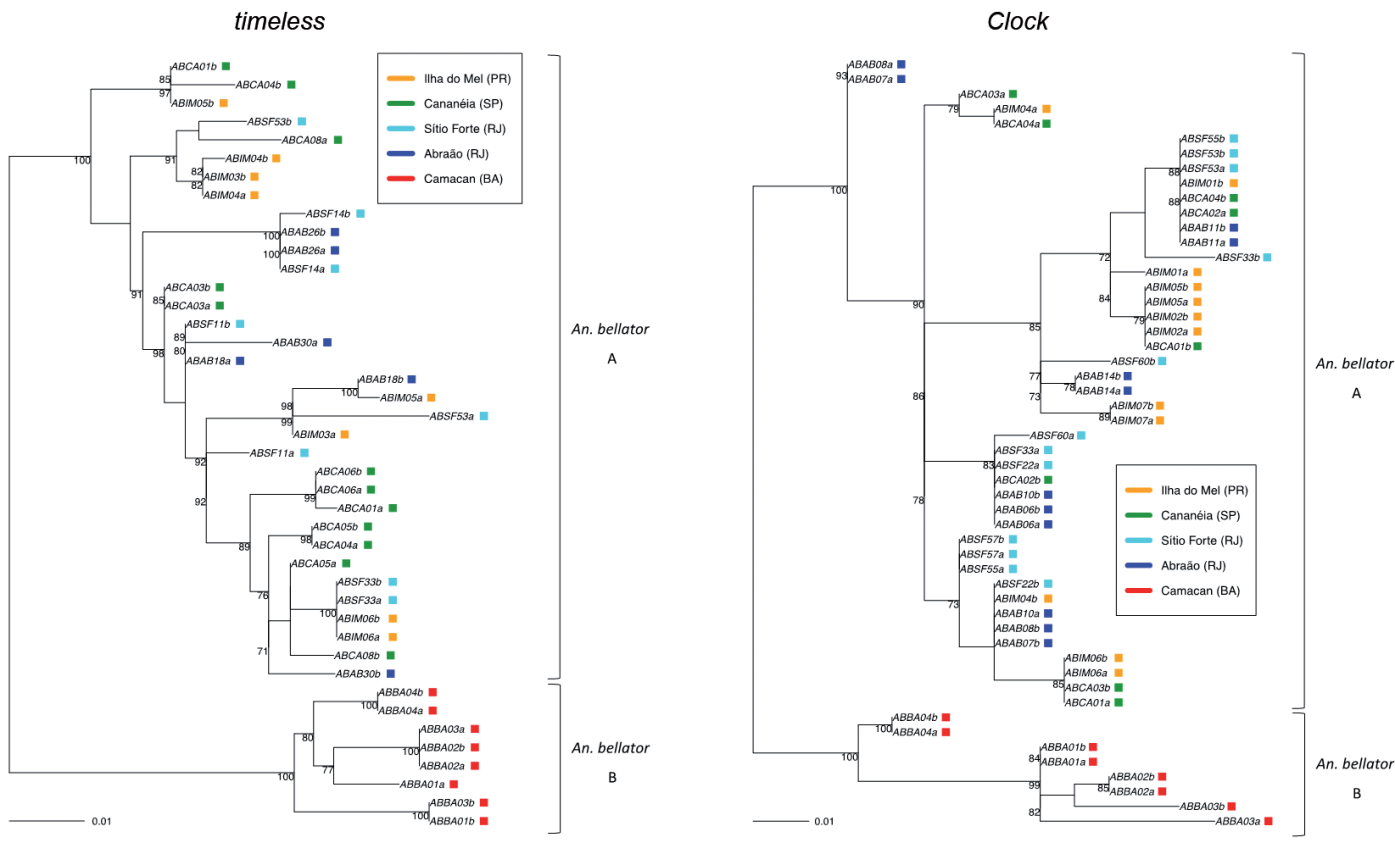
maximum likelihood trees of *Anopheles bellator timeless* and *Clock* sequences (TIM3e+G+I and HKY+G models, respectively). These phylogenies show that two structured groups were identified in the *An. bellator* populations from Brazil: *An. bellator* B occurs in the North-East Atlantic Rainforest (Camacan) and *An. bellator* A is found in the other more Southern Brazilian regions. Numbers on the nodes represent the percentage bootstrap values based on 1000 replications. Lowercase letters in the haplotype names specify the two alleles (A or B) obtained from each individual.

*Molecular data* - The *timeless* and *Clock* partial gene sequences from the five *An. bellator* populations were obtained by polymerase chain reaction (PCR), cloning and sequencing as described in de Rezende Dias et al.^14^ The primers used in the PCR reactions were those described in Rona et al.^10^ ^,^ ^11^ The sequencing was carried out in an ABI3130 DNA sequencer at Myleus Biotechnology. For each mosquito, at least eight clones were sequenced to mitigate PCR errors, and allow the identification of the two alleles. Consensus sequences representing the two alleles were generated. The individuals were classified as homozygotes when only one haplotype was observed among the eight sequences. Sequences were submitted to GenBank under the Accession Numbers: MG755641 - MG755734. An alignment of all *An. bellator* sequences for each gene is presented in Supplementary data (Figs. 1,2).

Sequences from the *timeless* gene fragment from *Anopheles* (*Kerteszia*) *laneanus* and *Anopheles* (*Kerteszia*) *cruzii* s.s. were used in order to verify the relationship of *An. bellator* samples analysed in this study with other closely related *Kerteszia* species. The sequences from *An. laneanus* were obtained by PCR, cloning and sequencing as described above and submitted to GenBank under the Accession Numbers: KT803975 - KT803978. The sequences from *An. cruzii* s.s. were those previously published by Rona et al. (10) (GenBank accession numbers FJ408732, FJ408735, FJ408746, FJ408786, FJ408788, FJ408813, FJ408816, FJ408832, FJ408736, FJ408738 for SC population and FJ408838, FJ408840, FJ408848, FJ408854, FJ408855, FJ408856, FJ408862, FJ408864 for BA population).

*DNA sequence analysis* - *Anopheles bellator* DNA sequences were aligned with MAFFT^19^ and phylogenetic trees were constructed for each gene under the Maximum Likelihood method using the *ape* and *phangorn* packages^20^ ^,^ ^21^ in R version 3.3.2.^18^ The best-fit substitution models HKY+G (*Clock*) and TIM3e+G+I (*timeless*) were selected following the AIC criterion using the modelTest function from *phangorn*.^21^ An additional *timeless* phylogenetic tree using the coding regions from *An. bellator*, *An. laneanus* and *An. cruzii* s.s. were also constructed (K80+G model) as described above.

DnaSP v5 software^22^ was used to calculate intra-population statistics, including nucleotide diversity and number of segregating sites, and also to ensure that all loci met the neutrality model by calculating the Tajima’s D.^23^ The P_RO_S_EQ_ v 2.91^24^ software was used to perform inter-population analyses, including the identification of fixed and shared polymorphisms and the generation of pair-wise estimates of population differentiation (e.g. *F*_*ST*_ , *Dxy* and *Da*).

## RESULTS AND DISCUSSION

*Population structure analysis* - A total of 94 sequences (two alleles from each *An. bellator* individual) were analysed for the two loci [Supplementary data (Tabela II)]. *Clock* and *timeless* fragment sequences were 164 bp and 368 bp long, respectively [Supplementary data (Figs 1,2)]. Polymorphism data from a variety of measures - *e.g.* nucleotide diversity (θ and π values) - showed that in general the *timeless* gene has lower genetic diversity compared with *Clock* [Supplementary data (Tabela II)], although the first has a bigger fragment size. None of the Tajima’s D results were significant, which means that there is no evidence of natural selection acting in any of the populations [Supplementary data (Tabela II)].

In all pairwise comparisons with the BA population, very high and significant *F*_*ST*_ values (ranging from ~ 0.62 to 0.67) were found, there were few shared polymorphisms and a large number of fixed differences (Table), some of which caused amino acid changes [Supplementary data (Figs 1,2)]. The average number of nucleotide substitutions per site (*Dxy*) and the number of net nucleotide substitutions per site between populations (*Da*) was also higher in the BA pairwise comparisons, confirming the *F*_*ST*_ measurements. The southern and southeastern populations exhibited limited differentiation, characterized by shared polymorphisms and no fixed differences.

**TABLE:** Genetic differentiation between all *Anopheles bellator* populations

Populations	Gene	Km	*F*_*ST*_	*Dxy*	*Da*	*Ss*	*Sf*
Abraão (RJ) *x* Sítio Forte (RJ)	*timeless*	10.89	-0.1039	0.0177	-0.0018	11	00
	Clock		-0.0157	0.0322	-0.0005	08	00
Ilha do Mel (PR) *x* Abraão (RJ)	*timeless*	496.5	0.0411	0.0215	0.0009	09	00
	Clock		0.1134	0.0404	0.0046	07	00
Ilha do Mel (PR) *x* Sítio Forte (RJ)	*timeless*	488.6	0.0695	0.0224	0.0016	11	00
	Clock		0.0904	0.0377	0.0034	08	00
Ilha do Mel (PR) *x* Cananéia (SP)	timeless	70.1	0.1147	0.0233	0.0027	10	00
	Clock		-0.0508	0.0376	-0.0019	10	00
Cananéia (SP) *x* Sítio Forte (RJ)	*timeless*	425.7	0.1158^*^	0.0221	0.0026	08	00
	Clock		0.0001	0.0364	0.0000	09	00
Cananéia (SP) *x* Abraão (RJ)	*timeless*	434.1	0.1311^*^	0.0222	0.0029	06	00
	Clock		0.0086	0.0383	0.0003	08	00
Camacan (BA) *x* Ilha do Mel (PR)	*timeless*	1449	0.6471^*^	0.0621	0.0402	00	07
	Clock		0.6482^*^	0.1168	0.0757	02	05
Camacan (BA) *x* Cananéia (SP)	*timeless*	1379	0.6694^*^	0.0622	0.0417	00	09
	Clock		0.6287^*^	0.1163	0.0731	02	05
Camacan (BA) *x* Abraão (RJ)	timeless	987.1	0.6722^*^	0.0628	0.0422	01	10
	Clock		0.6236^*^	0.1050	0.0655	02	03
Camacan (BA) *x* Sítio Forte (RJ)	*timeless*	990.1	0.6758^*^	0.0641	0.0434	00	09
	Clock		0.6562^*^	0.1105	0.0725	02	05

*F*_*ST*_: pair-wise estimates of population differentiation. The significance of *F*_*ST*_ values was evaluated by 1,000 random permutations (* p < 0.05). *Dxy*: average number of nucleotide substitutions per site between populations; *Da*: number of net nucleotide substitutions per site between populations.^29^ *Ss*: number of shared polymorphisms between the two populations. *Sf*: number of fixed differences between the two populations. Km: the approximated geographic distances between localities in km.

*Haplotype genealogies analysis* - The genealogical relationship of haplotypes was inferred by Maximum Likelihood for the two genes (Fig. 2). The resulting trees showed no clear separation between the sequences from PR, SP and RJ (henceforth called *An. bellator* A), however they clearly grouped the sequences from BA (henceforth called *Anopheles bellator* B) in a different cluster with high bootstrap values.

In order to verify the genealogical relationship between *An. bellator* A and B with other closely related *Kerteszia* species (*An. cruzii* s.s. and *An. laneanus*), a Maximum Likelihood tree was constructed using the coding regions of the *timeless* gene fragment, since the introns showed a substantial number of indels in the alignment [Supplementary data (Fig. 3)]. The resulting phylogenetic tree showed that *An. bellator* A and B are a monophyletic group, clustering them in a separated clade with high bootstrap value. The final topology was very similar to those from Foster et al.^25^ and Lorenz et al.^6^ using mitochondrial and genomic sequences: *An. cruzii* s.s. and *An. laneanus* are genetically closer when compared with *An. bellator*. An attempt was also made to compare *An. bellator Clock* sequences with those from different *Kerteszia* species available in the GenBank. Although, the same problem found in the *timeless* non-coding regions (indels) was also disclosed in the *Clock* alignment, but in a more challenging way, since more than 70% of the fragment is composed by an intron.

*Anopheles bellator A and B* - Two highly *An. bellator* structured groups (*An. bellator* A and B) were disclosed in this study. The population divergence between them is well supported by (i) fixed differences (Table), (ii) high *F*_*ST*_ values (average of 0.65) (Table), and (iii) it is the earliest divergent node in both phylogenetic trees with strong statistical support (Fig. 2). The results shown here are in agreement with a former study that used allozymes as genetic markers and showed that among all pairwise comparisons between Brazilian *An. bellator* samples, the highest genetic distances were found between Itaparica Island (BA) and the other more southern populations from Florianópolis (SC) and Cananéia (SP).^5^ However, the BA population (Camacan) used in this study is 300 Km away (to the south direction) from Itaparica Island (BA), which raises new questions like: Does *An. bellator* from Camacan belong to the same group as *An. bellator* from Itaparica Island? Also, are there additional structured *An. bellator* groups in the Atlantic Forest? A more extensive sampling across the geographic distribution of *An. bellator*, mainly focusing in Espírito Santo (ES) and BA, and the use of a larger number of molecular markers are desirable in order to better understand the evolutionary forces that establish and maintain the population structure of *An. bellator*. Accordingly, a number of other studies have shown the same separation pattern between northern and southern Atlantic Forest segments in many fauna (including *An. cruzii* s.s.) and flora groups, which converge around northern ES and southern BA, suggesting a common vicariant event.^10^ ^,^ ^11^ ^,^ ^26^

The high genetic differentiation observed between BA and the more southern populations might also be explained by the geographic distance between them (at least 980 Km away) (Table). However, we would expect higher *F*_*ST*_ values in the pairwise comparisons between PR and SP, with the two populations from RJ (average *F*_*ST*_ of only 0.07), which do not have a negligible distance apart (~ 500 km). Even so, among the southern and southeastern populations, we found a widespread lack of significant differentiation.

This study uncovers two structured *An. bellator* groups in the Brazilian Atlantic Forest: *An. bellator* A is widespread in Southern and Southeastern regions, and *An. bellator* B is found in the north-east Brazil. Interestingly, in spite of the fact that malaria cases are accounted for yearly in BA, *An. bellator* has never been viewed as a malaria vector in this state^27^ ^,^ ^28^ as it is for the Southern Brazilian regions.^2^ So, future studies would be valuable in order to differentiate the two *An. bellator* groups regarding malaria susceptibility.
