# Supplementary material for: Novel molecular evidence of population structure in Anopheles (Kerteszia) bellator from Brazilian Atlantic Forest
Source: Mem Inst Oswaldo Cruz. 2019 May 13;114:e180598. doi: 10.1590/0074-02760180598 (PMC6516739; doi:10.1590/0074-02760180598)
Supplement: Supplementary file 1 [file 1678-8060-mioc-114-e180598-s.pdf]

TABLE I

Anopheles bellator field collections

Individual ID	Country	State	Locality	latitude	longitude	Year	Month	Sex	Source
ABAB06	Brazil	Rio de Janeiro (RJ)	Abraão, Ilha Grande, Angra dos Reis	-23.134761	-44.170019	2012	August	M	larvae
ABAB07	Brazil	Rio de Janeiro (RJ)	Abraão, Ilha Grande, Angra dos Reis	-23.134761	-44.170019	2012	August	M	larvae
ABAB08	Brazil	Rio de Janeiro (RJ)	Abraão, Ilha Grande, Angra dos Reis	-23.134761	-44.170019	2012	August	M	larvae
ABAB10	Brazil	Rio de Janeiro (RJ)	Abraão, Ilha Grande, Angra dos Reis	-23.134761	-44.170019	2012	August	M	larvae
ABAB11	Brazil	Rio de Janeiro (RJ)	Abraão, Ilha Grande, Angra dos Reis	-23.134761	-44.170019	2012	August	M	larvae
ABAB14	Brazil	Rio de Janeiro (RJ)	Abraão, Ilha Grande, Angra dos Reis	-23.134761	-44.170019	2012	August	M	larvae
ABAB18	Brazil	Rio de Janeiro (RJ)	Abraão, Ilha Grande, Angra dos Reis	-23.134761	-44.170019	2012	August	F	larvae
ABAB26	Brazil	Rio de Janeiro (RJ)	Abraão, Ilha Grande, Angra dos Reis	-23.134761	-44.170019	2012	August	F	larvae
ABAB30	Brazil	Rio de Janeiro (RJ)	Abraão, Ilha Grande, Angra dos Reis	-23.134761	-44.170019	2012	August	F	larvae
ABBA01	Brazil	Bahia (BA)	Camacan	-15.391917	-39.565167	2015	March	F	adult
ABBA02	Brazil	Bahia (BA)	Camacan	-15.391917	-39.565167	2015	March	F	adult
ABBA03	Brazil	Bahia (BA)	Camacan	-15.391917	-39.565167	2015	March	F	adult
ABBA04	Brazil	Bahia (BA)	Camacan	-15.391917	-39.565167	2015	March	F	adult
ABCA01	Brazil	São Paulo (SP)	Cananéia	-25.016667	-47.916667	2003	August	F	adult
ABCA02	Brazil	São Paulo (SP)	Cananéia	-25.016667	-47.916667	2003	August	F	adult
ABCA03	Brazil	São Paulo (SP)	Cananéia	-25.016667	-47.916667	2003	August	F	adult
ABCA04	Brazil	São Paulo (SP)	Cananéia	-25.016667	-47.916667	2003	August	F	adult
ABCA05	Brazil	São Paulo (SP)	Cananéia	-25.016667	-47.916667	2003	August	F	adult
ABCA06	Brazil	São Paulo (SP)	Cananéia	-25.016667	-47.916667	2003	August	F	adult
ABCA08	Brazil	São Paulo (SP)	Cananéia	-25.016667	-47.916667	2003	August	F	adult
ABIM01	Brazil	Paraná (PR)	Paranaguá, Ilha do Mel	-25.516167	-48.333972	2014	January	F	larvae
ABIM02	Brazil	Paraná (PR)	Paranaguá, Ilha do Mel	-25.516167	-48.333972	2014	January	F	larvae
ABIM03	Brazil	Paraná (PR)	Paranaguá, Ilha do Mel	-25.516167	-48.333972	2014	January	F	larvae
ABIM04	Brazil	Paraná (PR)	Paranaguá, Ilha do Mel	-25.516167	-48.333972	2014	January	F	larvae
ABIM05	Brazil	Paraná (PR)	Paranaguá, Ilha do Mel	-25.516167	-48.333972	2014	January	F	larvae
ABIM06	Brazil	Paraná (PR)	Paranaguá, Ilha do Mel	-25.516167	-48.333972	2014	January	M	larvae
ABIM07	Brazil	Paraná (PR)	Paranaguá, Ilha do Mel	-25.516167	-48.333972	2014	January	?	larvae
ABSF11	Brazil	Rio de Janeiro (RJ)	Sítio Forte, Ilha Grande, Angra dos Reis	-23.117986	-44.274947	2012	August	F	larvae
ABSF14	Brazil	Rio de Janeiro (RJ)	Sítio Forte, Ilha Grande, Angra dos Reis	-23.117986	-44.274947	2012	August	F	larvae
ABSF22	Brazil	Rio de Janeiro (RJ)	Sítio Forte, Ilha Grande, Angra dos Reis	-23.117986	-44.274947	2012	August	F	larvae
ABSF33	Brazil	Rio de Janeiro (RJ)	Sítio Forte, Ilha Grande, Angra dos Reis	-23.117986	-44.274947	2012	August	F	larvae
ABSF53	Brazil	Rio de Janeiro (RJ)	Sítio Forte, Ilha Grande, Angra dos Reis	-23.117986	-44.274947	2012	August	F	larvae
ABSF55	Brazil	Rio de Janeiro (RJ)	Sítio Forte, Ilha Grande, Angra dos Reis	-23.117986	-44.274947	2012	August	F	larvae
ABSF57	Brazil	Rio de Janeiro (RJ)	Sítio Forte, Ilha Grande, Angra dos Reis	-23.117986	-44.274947	2012	August	F	larvae
ABSF60	Brazil	Rio de Janeiro (RJ)	Sítio Forte, Ilha Grande, Angra dos Reis	-23.117986	-44.274947	2012	August	?	larvae

This file includes the field collection information for each sample used in this study as follow: individual ID, the identity for each sample; country, state, locality, latitude and longitude, collection sites details for each collection point; year and month, collection dates; source, if the specimens were collected as adults or larvae. The collected immatures were reared in the laboratory until become adults. In Cananéia (SP) and Camacã (BA), only adult females captures were performed; in the other three localities, only immatures captures were executed. M: males; F: females; ?: undetermined sex.

TABLE II

Polymorphisms of all *Anopheles bellator* populations

Population	Gene	N	S	θ	π	D_T
Abraão (RJ)	timeless	06	22	0.0266	0.0258	-0.1963
	Clock	12	11	0.0224	0.0288	1.1698
Camacan (BA)	timeless	08	17	0.0178	0.0199	0.6018
	Clock	08	15	0.0375	0.0354	-0.2837
Cananéia (SP)	timeless	12	18	0.0174	0.0194	0.5138
	Clock	08	12	0.0285	0.0346	1.0643
Ilha do Mel (PR)	timeless	08	20	0.0230	0.0243	0.2898
	Clock	12	11	0.0224	0.0311	1.6025
Sítio Forte (RJ)	timeless	08	17	0.0215	0.0197	-0.4378
	Clock	12	11	0.0278	0.0330	0.7890

N: number of DNA sequences of each population; S: number of polymorphic (segregating) sites; θ: nucleotide diversity based on the total number of mutations; π: nucleotide diversity based on the average number of pair-wise differences; DT: Tajima's D,(23) based on the total number of mutations. In no cases were Tajima's D-values significant ($p > 0.10$ in all cases).

DNA sequences alignments of the timeless and *Clock* gene fragments of all individuals from *Anopheles bellator* populations analysed. The introns are presented in the dark grey regions. Dots represent the identity of the first nucleotide sequence. The three non-synonymous changes on the *timeless* gene are highlighted in grey at the positions 18, 21 and 25, inside the first exon fragment. The timeless gene alignment has two continuous pages. The first one includes the first 180 sites, and the second includes the rest of the fragment. ABBA: Camacã (BA); ABAB: Abraão (RJ); ABSF: Sítio Forte (RJ); ABCA: Cananéia (CA); ABIM: Ilha do Mel (PR)

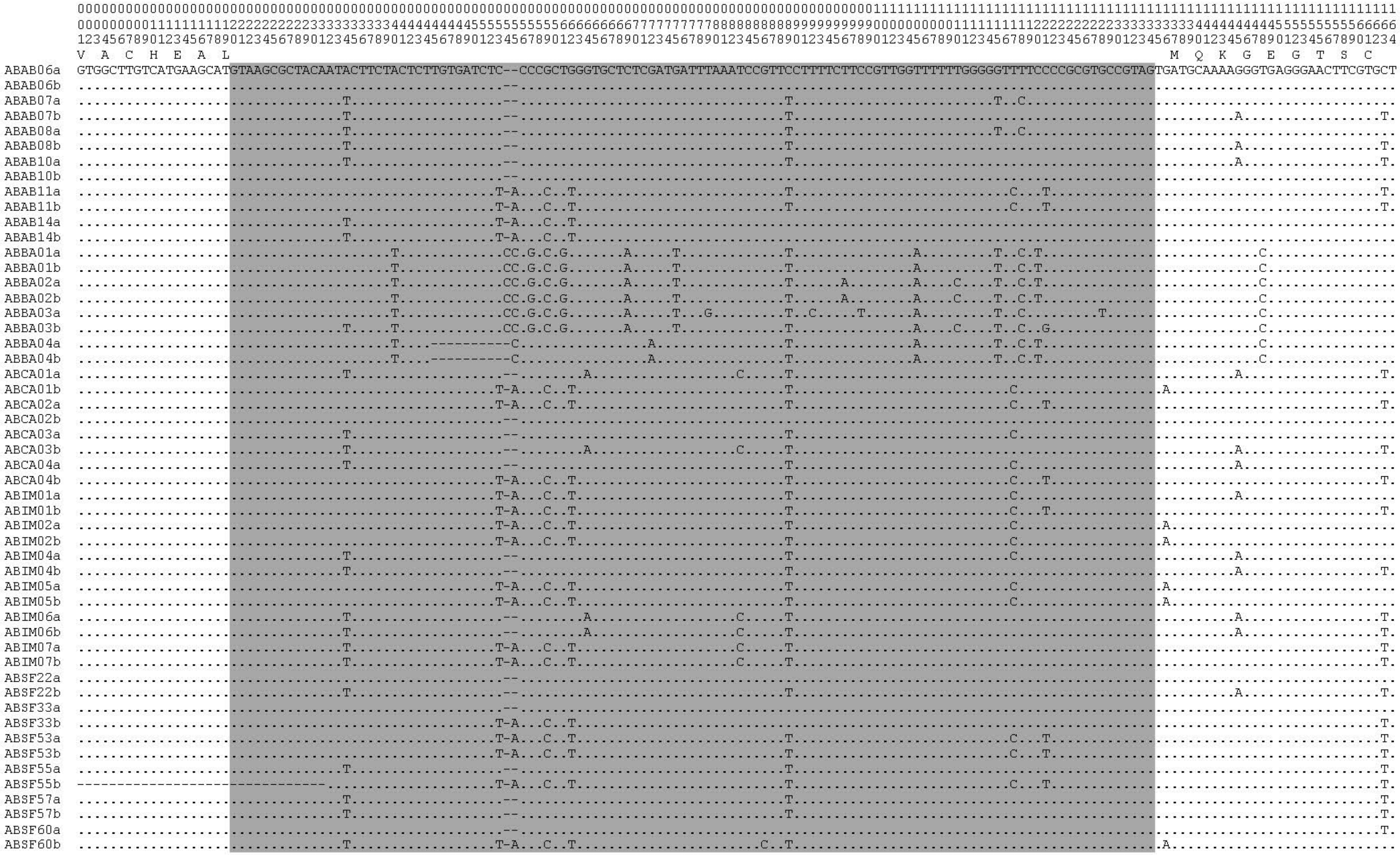

Fig. 1: alignment of the DNA sequences from the *Clock* gene fragment.

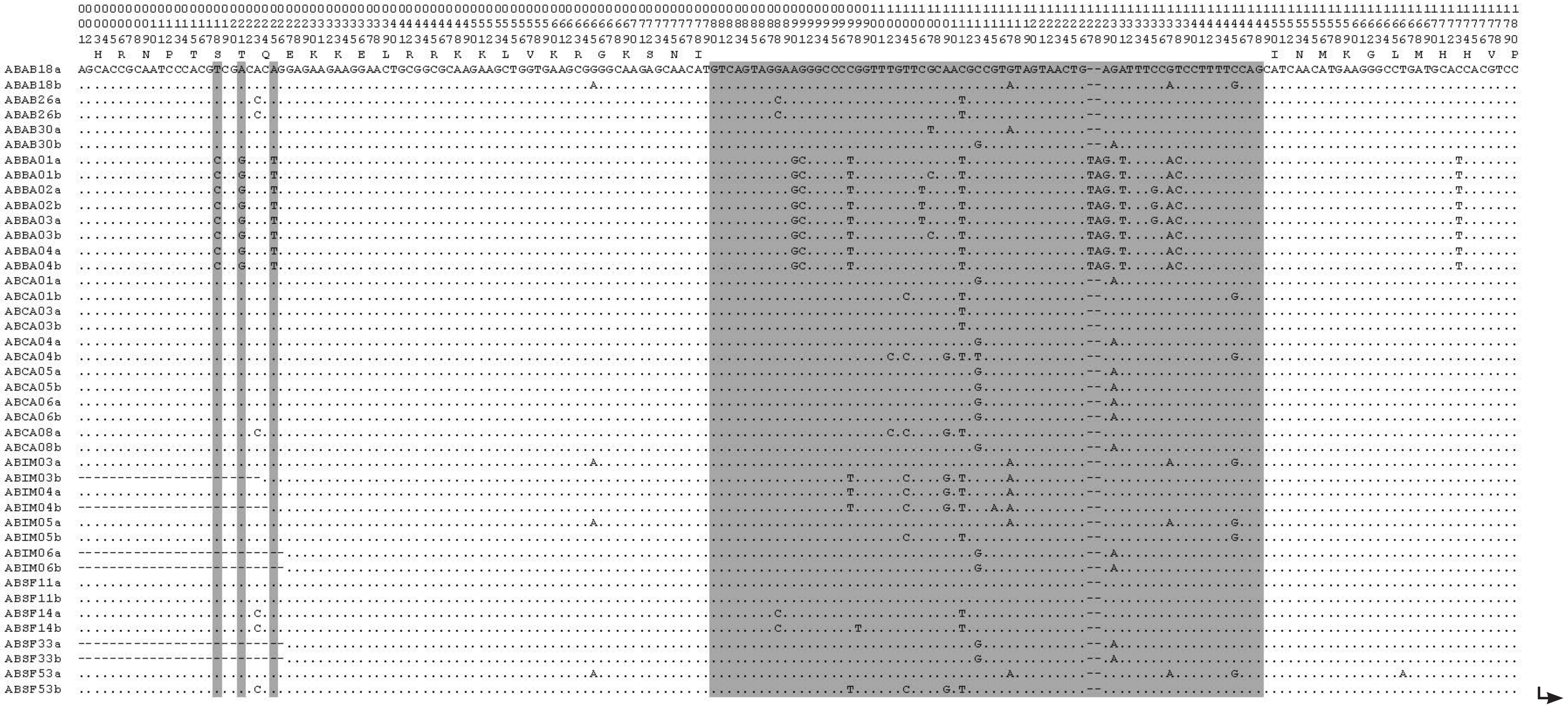
ABAB18a
ABAB18b
ABAB26a
ABAB26b
ABAB30a
ABAB30b
ABBA01a
ABBA01b
ABBA02a
ABBA02b
ABBA03a
ABBA03b
ABBA04a
ABBA04b
ABCA01a
ABCA01b
ABCA03a
ABCA03b
ABCA04a
ABCA04b
ABCA05a
ABCA05b
ABCA06a
ABCA06b
ABCA08a
ABCA08b
ABIM03a
ABIM03b
ABIM04a
ABIM04b
ABIM05a
ABIM05b
ABIM06a
ABIM06b
ABSF11a
ABSF11b
ABSF14a
ABSF14b
ABSF33a
ABSF33b
ABSF53a
ABSF53b

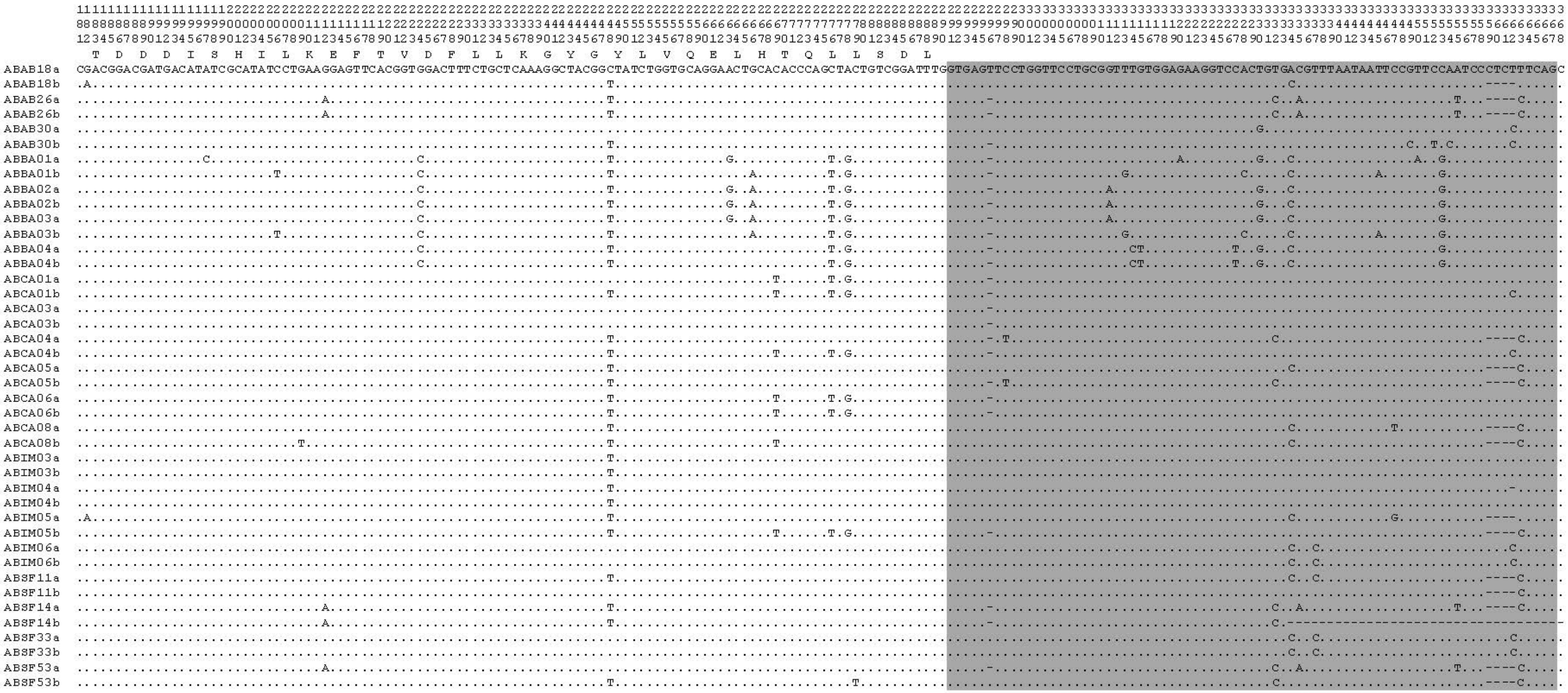

Fig. 2: alignment of the DNA sequences from the *timeless* gene fragment.

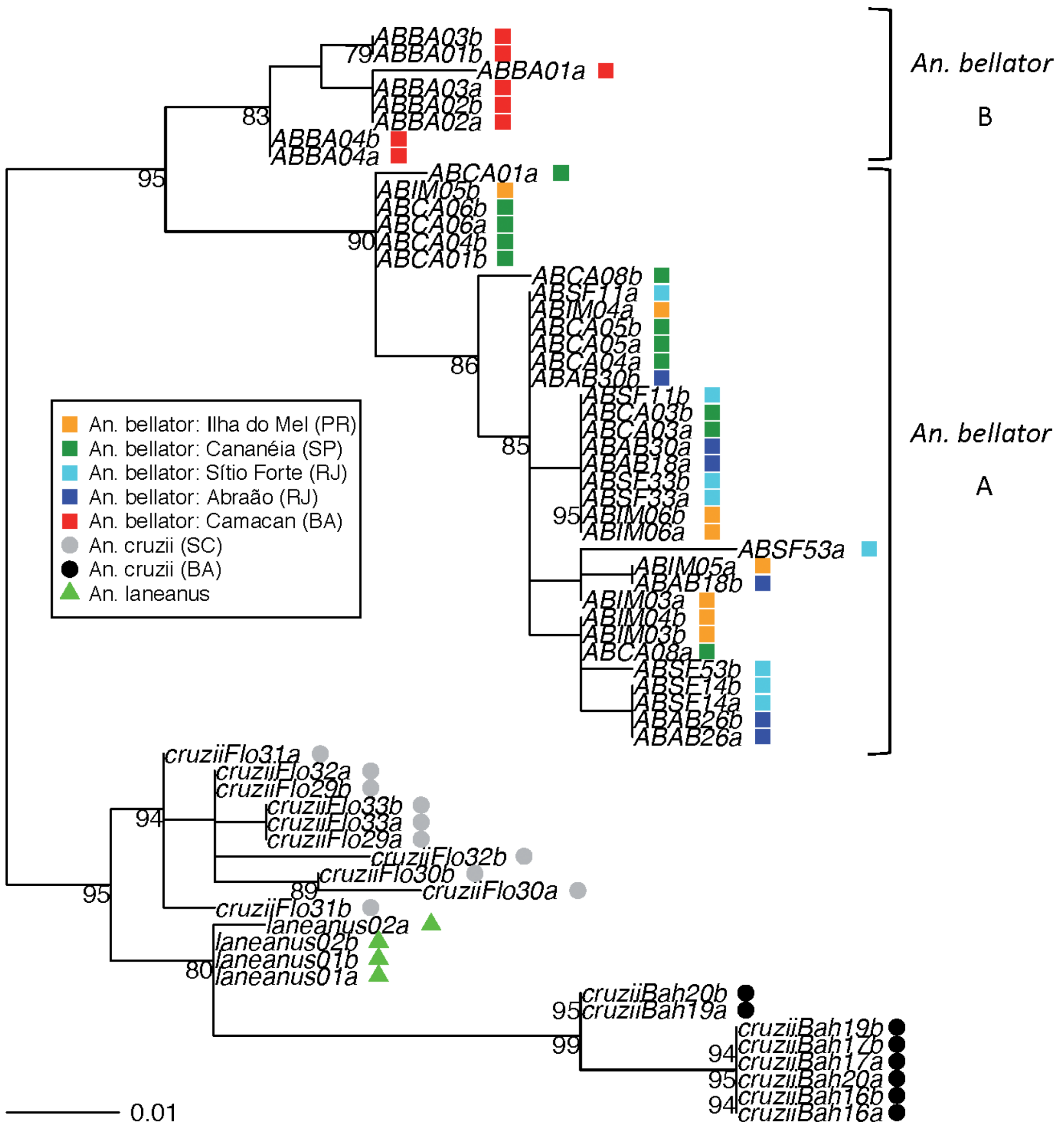

Fig. 3: maximum likelihood tree of *Anopheles bellator*, *An. cruzii* s.s. and *An. laneanus timeless* sequences (K80+G model). The *timeless* gene phylogeny clearly shows the monophyly between *An. bellator* A and B when compared with other closely related Kerteszia species. Numbers on the nodes represent the percentage bootstrap values based on 1000 replications. Lowercase letters in the haplotype names specify the two alleles (A or B) obtained from each individual.
